# Development of an eHealth programme for self-management of persistent physical symptoms: a qualitative study on user needs in general practice

**DOI:** 10.1186/s12875-021-01380-5

**Published:** 2021-02-07

**Authors:** Mette Trøllund Rask, Pernille Ravn Jakobsen, Jane Clemensen, Marianne Rosendal, Lisbeth Frostholm

**Affiliations:** 1 Research Clinic for Functional Disorders and Psychosomatics, Aarhus University Hospital, Nørrebrogade 44, 8000 Aarhus, Denmark; 2 University College Southern Denmark, Lembckesvej 3-7, 6100 Haderslev, Denmark; 3 Hans Christian Andersen Children’s Hospital, Odense University Hospital, Kløvervænget 23, 5000 Odense, Denmark; 4 Research Unit for General Practice, Bartholins Allé 2, 8000 Aarhus, Denmark; 5 Department of Clinical Medicine, Aarhus University, Nørrebrogade 44, 8000 Aarhus C, Denmark

**Keywords:** eHealth, General practice, Persistent physical symptoms, Medically unexplained symptoms, Self-management, Behaviour change wheel

## Abstract

**Background:**

Persistent physical symptoms (PPS) are estimated to be present in 17% of patients in general practice. Hence, general practitioners (GPs) play a key role in both the diagnostic assessment and the management of PPS. However, research indicates a need to improve their strategies to support self-help in patients, and eHealth tools may serve as an opportunity. This study aimed to explore patients’ and GPs’ needs related to self-management of PPS. The study was designed to inform the future development of eHealth interventions in this field.

**Methods:**

This qualitative study was based on 20 semi-structured interviews (6 GPs and 14 patients with PPS). Interviews were audiotaped, transcribed verbatim and analysed through a five-step thematic analysis approach. First, we conducted an inductive analysis to identify and explore emerging subthemes. Second, using a deductive mapping strategy, we categorised the derived subthemes according to the COM-B behaviour change model and its three domains: capability, opportunity and motivation.

**Results:**

We identified eleven subthemes in the patient interviews and seven subthemes in the GP interviews. Several unmet needs emerged. First, we identified a need to consider PPS early in the illness trajectory by taking a bio-psycho-social approach. Second, both patients and GPs need better skills to manage uncertainty. Third, hope is important for the patients. Fourth, patients need guidance from their GP in how to self-manage their PPS.

**Conclusions:**

This study provides important insight into key issues and needs related to capability, opportunity and motivation that should be addressed in the design of future eHealth self-management interventions targeting patients with PPS in general practice in order to support and improve care.

**Supplementary Information:**

The online version contains supplementary material available at 10.1186/s12875-021-01380-5.

## Background

Patients with persistent physical symptoms (PPS), such as pain, dizziness and fatigue, which cannot be attributed to a specific well-defined disease, are frequently seen in general practice and secondary care. The frequent attendance of many patients with PPS means that the medical costs for this group of patients are ranked among the highest of all patient groups [1, 2]. PPS are estimated to be present in 10% of adults in the general population according to standardised interviews and in 17% in patients attending general practice according to questionnaire screening [3, 4]. In general, PPS are associated with high morbidity, distress in patients and their families, high healthcare costs and high risk of loss of work capacity [4, 5].

PPS represent a spectrum of severity, ranging from mild symptoms to severe and chronic disorders [6]. PPS is a newly introduced term for subjective complaints that are not verifiable by clinical or paraclinical findings [7]. Traditionally, the term *medically unexplained symptoms* (MUS) has been used. However, in recent research, this term has been argued to be misleading, and it is unhelpful to patients with PPS [7–9]. A recent survey among the general population suggested *persistent physical symptoms* as the most appropriate and acceptable term [10]. Consequently, we have chosen this term in this paper.

General practice is the first point of entry to the healthcare system in many European countries. Hence, GPs play a central role in both the diagnosis and management of patients with PPS [11]. A qualitative study of GP consultations on PPS reported a mismatch between the GP’s agenda and the patient’s agenda. Patients have indicated that the GP may evoke uncomfortable feelings in them during the consultation, without providing a specific management plan for their symptoms. In general, patients often found the GP to be inadequately prepared for the consultation, and they perceived the GP to be prejudiced [12]. In line with this, GPs often experience patients with PPS as burdensome and difficult to treat, and negative attitudes towards these patients are found among GPs [13, 14]. GPs struggle with the incongruence between the patients’ persistent symptom presentations and the explanatory models for biomedical disease [15]. The lack of adequate treatment strategies for patients with PPS is a major problem, as early management of PPS in general practice may prevent the symptoms from becoming chronic and disabling, which could help reduce the burden on patients, clinicians and society [16].

Technological advances allow for new delivery methods and provision of treatment services that so far have been available in specialised care settings only [17, 18]. This may provide an opportunity to deal with some of the challenges of PPS in general practice. During the past decade, a wide range of free or low-cost apps and online resources have been made publicly available, and many of these offer mindfulness training, stress reduction and other self-help strategies. However, this market is mostly unregulated and often unguided. Several self-help interventions and eHealth programmes have recently been evaluated in two systematic reviews of randomised controlled trials focusing on specific symptoms or disorders. These reviews indicate that such interventions may contribute to symptom alleviation and increase quality of life [19, 20]. However, no self-help interventions have been developed for PPS to target general symptoms (rather than specific disorders) at an early point in time.

We defined a research programme with the overall objective to develop an online self-help intervention tailored to patients with early signs of PPS in general practice. A major challenge of eHealth solutions is to make it beneficial and easy to use for both healthcare providers and patients; otherwise implementation is likely to fail [21, 22]. Furthermore, little is known about how such eHealth interventions should be designed to meet the needs of both patients with PPS and GPs. Therefore, as a first step of the research programme, the aim of the present study was to identify patients’ and GPs’ needs related to self-management of PPS prior to the development of a GP-prescribed eHealth intervention for PPS.

## Methods

### Design

We conducted a qualitative study based on semi-structured individual interviews with GPs and patients with PPS in the Region of Southern Denmark. The COM-B model, a framework enabling a systematic evaluation of behavioural aspects, guided the development of the interview guide and the conduction of the interviews and the analyses. The COM-B model proposes three interacting domains for behaviour change to occur: *Capability, Opportunity* and *Motivation* [23]. *Capability* is defined as the individual’s psychological and physical capacity to engage in the activity concerned; this includes having the necessary knowledge and skills. *Opportunity* is defined as all factors outside the individual that enable the behaviour or prompt it. *Motivation* is defined as all brain processes that direct behaviour, e.g. goals, conscious and analytical decision-making, habitual processes and emotional response.

### Participants

We applied a purposeful sampling strategy seeking to include GPs representing different age groups, genders, practice types, geographical locations and with different levels of knowledge of and interest in PPS and attitudes towards eHealth [24]. One of the authors (MR) is very familiar with the general practices in the region through her administrative position, and she selected and invited the GPs for participation.

Patients who participated in the semi-structured interviews represented a convenience sample that was invited by the participating GPs and included by a researcher. To be eligible for inclusion, patients should 1) be aged 18–65 years, 2) suffer from PPS according to their GP and report to be ‘somewhat bothered’ by at least four symptoms (each symptom scored ≥2 on a Likert scale from 0 (‘Not bothered at all’) to 4 (‘Bothered a lot’)) as measured by the Bodily Distress Syndrome (BDS) checklist [25] and 3) be able to speak and understand Danish. Exclusion criteria were severe mental disorder and sick leave of more than eight consecutive weeks.

Patients were verbally informed about the study by their GP. They received an information letter and were asked for permission to be contacted by a researcher. Patients who agreed were asked to complete the BDS checklist at home. Subsequently, the patients were contacted by phone by the second author and received further information about the study. If they gave verbal consent to participate, they were screened according to the inclusion criteria; if these were met, an interview was scheduled. The included patients signed a consent form prior to the interview.

### Data collection procedure

#### Interview guides

The second author (PRJ), who is formally trained in qualitative methods and participatory design, conducted field studies. These included observation of patient-physician encounters in the included general practices (seven working days in total) in order to get further insight into the field of general practice and to inform the interview guides. The development of the interview guides was inspired by the COM-B model, and they were pilot tested and revised prior to the interviews.

The GP interviews focussed on how the GPs experienced and managed patients with PPS. This approach was taken to reveal capability, opportunity and motivation, and to identify barriers and enablers in providing self-help through an eHealth intervention. The patient interviews focussed on the patients’ descriptions of their symptoms, their experience of having PPS, and their capability, opportunity and motivation for self-management in order to identify barriers and enablers that should be considered in the development of an eHealth intervention (see Supplementary file 1 for interview guides).

#### Semi-structured interviews

The interviews were conducted by PRJ. The GPs were interviewed in their practice, and the patients were interviewed in their homes (*n* = 13) or at their workplace (*n* = 1). During all interviews, only the participant and the interviewer were present, and all interviews were conducted in a quiet and uninterrupted environment. The interviews lasted 37–87 min, with an average length of 54 min.

### Analyses

All interviews were recorded and transcribed verbatim, and additional field notes were taken down immediately after each interview. Interview transcripts and field notes were anonymised and stored in accordance with the Danish data protection regulations. The interview data were subject to a thematic analysis by the research team, and the analysis was guided by Braun and Clarke’s five-step framework [26]. The primary analysis was based on an inductive approach and was done separately for patients and GPs in an iterative process.

The cross-disciplinary research team represented different academic backgrounds (Master of Health Sciences, GP, psychologist and Master of Science in Nursing). All authors hold a PhD degree and are experienced in the fields of general practice, PPS and/or qualitative methods. Three of the authors (PRJ, MTR and LF) performed the first three steps of the analysis, including the coding of meaningful text units. The codes and the emerging subthemes were discussed among the three coders at two consensus meetings (Table 1).

**Table 1:** The five steps of the thematic analysis

Step	Description
I	Familiarisation with data (PRJ, MTR, LF)
II	Inductive coding of data (PRJ, MTR and LF coded data independently and compiled an initial list of preliminary ideas about subthemes)
III	Discussion of codes and consensus reached on emerging subthemes (PRJ, MTR, LF)
IV	Exploration and refinement of subthemes, including similarities and differences between interviews (PRJ, MTR, LF, JC and MR)
V	Review and final revision of agreed subthemes and deductive mapping to the COM-B model (PRJ, MTR, LF, JC and MR)

No new subthemes emerged during the coding process of the final GP and patient interviews, indicating that data saturation was reached. The subthemes were further explored and refined by the entire research team. Finally, the identified subthemes were mapped to the COM-B framework in a deductive process in order to allow the expressed needs of the GPs and the patients to be translated into the development of the self-help intervention [23].

## Results

### Participant characteristics

During January and February 2019, six GPs (four females, two males) from four general practices were invited to participate in the study and agreed to participate. The age of the GPs ranged from 46 to 62 years (mean: 52.8 years). All participating GPs were affiliated with a general practice that was either a partnership practice or a solo practice forming part of a formal collaboration. One practice was situated in a rural area, and three were located in urban areas. Two of the GPs took a special interest in PPS, and a third GP had a special interest in eHealth/digital solutions.

From January to March 2019, 16 patients were invited to participate in the study. Two patients were excluded due to severe mental disorders. In total, 14 patients (ten females, four males) were included. The age of the patients ranged from 20 to 58 years (mean: 35 years). The number of ‘somewhat bothering’ symptoms on the BDS checklist ranged from 5 to 25 (mean: 13.4), and the symptom duration ranged from 2.5 to 108 months (mean: 29 months). Ten patients were employed, one was unemployed, and three were on sick leave (less than 8 weeks).

### Patient and GP interviews

In the analysis, 11 subthemes were identified in the patient interviews, and seven subthemes were identified in the GP interviews. The subthemes were mapped to the three overriding themes represented by the COM-B domains (Table 2).

**Table 2:** Subthemes from the patient and GP interviews mapped to the domains of capability, opportunity and motivation (COM-B model). Italics indicate the identified subthemes

COM-B domain	Patient themes	GP themes
**Capability**	**Physical** *Jungle of management strategies*	**Physical** *Missing biomedical reassurance*
	**Psychological** *Understanding causes of PPS* *Dealing with uncertainty* *Timing in bringing PPS into play*	**Psychological** *Dealing with uncertainty* *Timing in bringing PPS into play*
**Opportunity**	**Physical** *Time constraints*	**Physical** *Lack of resources*
	**Social** *Shift in social roles due to PPS* *GP as a discussion partner*	**Social** *Cultural norms in the healthcare system*
**Motivation**	*Guidance from the GP* *Need for hope* *Tracking of symptoms* *Easy access to self-help strategies*	*Fewer and beneficial consultations* *Better prepared for consultations*

### Themes identified in the patient interviews

The patient interviews provided information about how the patients experienced PPS. Furthermore, the three domains in the COM-B model were used to guide the identification of the patients’ needs in relation to self-management of their symptoms.

### Domain 1: capability

Capability was explored in order to establish whether the patients felt physically and psychologically capable of self-managing their PPS.

### Physical capability

#### Jungle of management strategies

The patients described a general lack of skills and knowledge about how to manage their symptoms. They applied various strategies in search for symptom relief; some by avoidance of certain situations and withdrawal from social relations, others by making use of various community-based initiatives or public mental health services such as psychologists, physiotherapists, massage therapist, osteopaths, acupuncturists or chiropractors. Others participated in mindfulness classes, yoga sessions or exercised in hot water.

Many patients described that friends and family came to their aid with advice and support with the best intentions, but these often contributed to uncertainty and confusion. Instead, patients expressed a need for competent help to navigate the ‘jungle of management strategies’. A female patient found her GP and physiotherapist to be very helpful as they guided her in which specific actions she could take, and how she could avoid putting strain on herself. However, many patients felt that the advice and support received from their GP was not sufficient; it was too general and did not meet their specific needs. As one woman stated:

*“My GP tells me to pay attention to my bodily sensations, but it is not clear to me what I am supposed to be attentive to”* (P1, female, aged 56)*.*

### Psychological capability

#### Understanding causes of PPS

The majority of patients described psychological distress as a starting point for their symptoms. Distress could arise due to varying circumstances in the patient’s life, such as heavy workload, raising children or a mid-life crisis, but it could also be caused by serious life events, such as divorce, an accident or the loss of a loved one. Many patients recognised distress as part of their problem, but they expressed uncertainty as to whether their symptoms were caused by distress or by an underlying and not yet diagnosed physical disease. The latter was a cause of fear.

Although the majority of patients experienced psychological aspects to contribute to their symptoms, only few patients experienced that a multi-factorial explanation for their symptoms was addressed by their GP. Instead, the focus was on physical aspects, and the patients were often referred to further physical investigations as described by one of the participants:

*“It was a tough period for me. Small kids and busy at work. Suddenly, I felt like my legs could not carry me anymore. My GP referred me to the hospital, but the neurologist could not find any explanation. Later, I realised myself the causality between stress and physical symptoms”* (P6, female, aged 37).

One participant suggested that it would have been more helpful if the GP had focused on how he was doing in life in general, instead of exploring his stomach symptoms only:

*“I was worried; a new-born baby, a new house, the symptoms from my stomach and being unsure whether I would be able to keep my job, you know, a jumble of thoughts. And then I think that my GP could have helped me if she had asked how I was actually doing instead of only focusing on the symptoms from my stomach”* (P14, male, aged 33).

#### Dealing with uncertainty

Despite the openness to a multi-factorial explanation for the symptom presentations, the uncertainty of symptom origin and the fear of an underlying physical disease made many patients express a need for more thorough tests and investigations. A woman in her early thirties with persistent low back pain described:

*“I really need some more physical investigations to be sure that nothing severe is wrong with me, and if they show that everything is fine, we can take it from there”* (P9, female, aged 32).

Yet, when referrals to a specialist revealed no abnormal findings, some patients still felt insecure and started to worry about whether the physicians performing the investigations had been sufficiently careful. A woman who had been referred to a gynaecologist due to abdominal symptoms shared her thoughts:

*“I keep on thinking about whether the gynaecologist was thorough enough. He was very busy that day, and there were a lot of patients in the waiting room”* (P11, female, aged 38).

Even though some of the patients did not believe that an underlying disease was the cause of their symptoms, they requested physical investigations to be on the safe side prior to bringing up other explanations, such as distress, in the consultations. To some patients, the approach seemed to be ‘the more physical investigations, the better’*.* Yet, some patients also described an ambivalence between being on the safe side and being worried when referred for physical investigations.

Whether or not extensive physical tests and investigations were done on the patient’s request or the GP’s advice, normal findings seemed to contribute neither to an improved symptom understanding nor to reassurance or reduced fear.

#### Timing in bringing PPS into play

In the recruitment of patients for the study, the GPs had to explain about the study aim and PPS. For some of the patients, this was the first time they were introduced to PPS. Some of the patients experienced this as a revelation and argued that it would have been helpful if PPS had been provided as an alternative explanation concurrently to the physical investigations. A female participant in her late fifties said:

*“My GP could have told me that of course we have to do investigations in order to exclude any critical condition, but maybe you should instead learn to manage your symptoms and understand what might cause them”* (P8, female, aged 58).

By bringing PPS into play earlier, the patients believed that they would be more willing to accept PPS as a reasonable explanation if the physical investigations showed no abnormalities and provided no explanations. However, the timing was thought to be of utmost importance; some of the patients argued that if the GP had introduced PPS as a possible cause for their symptoms at the first encounter and prior to the physical investigations, they would have rejected it. They would first need to be sure that nothing serious was wrong with their body:

*“If it had been introduced to me at the first time I had symptoms from my heart after my father’s death of a heart attack, I don’t think that I would have been prepared to accept it. At that time, I really needed to be sure that there was nothing wrong with my heart”* (P8, female, aged 58).

Thus, the patients expressed a need for the GP to bring PPS into play at an earlier time in their symptom trajectory, yet not without first excluding serious physical disease.

### Domain 2: opportunity

Physical opportunity was explored to understand how the environment and the available resources in general practice affected the patients’ self-management of PPS. Social opportunity was explored to understand how the cultural norms and the interpersonal relations affected the experience and self-management of PPS.

### Physical opportunity

#### Time constraints

In Denmark, a GP has an average of 10 min for each patient, which leads some GPs to address only one symptom per consultation. This may be explicitly expressed to the patients, who are asked to present only one symptom and book another consultation if more symptoms need to be addressed. Many of the included patients found the encounters due to diffuse persistent symptoms challenging because of the time-constrained consultations with their GP. A woman found GPs to be overly focused on medications and continued:

*“I wish that I could visit my GP every two weeks, without any specific purpose besides having a deep and meaningful conversation. As it is now, there is only time for a superficial chat”* (P2, female, aged 32).

### Social opportunity

#### Shift in social roles due to PPS

The patients described how their role in relation to their social network and their families had changed after the symptoms started. They described a feeling of guilt as they were unable to be the friend, mother, father, wife or husband they used to be. A man with one child described it this way:

*“I want to be there for my little son, but when I get dizzy, I have to go into the bedroom and rest, and I really find that hard to accept”* (P14, male, aged 33).

Several patients described how the symptoms had forced them to withdraw from daily chores and instead leave these tasks to their spouse and children. A middle-aged woman living with her husband and a son explained:

*“I am not able to do anything anymore. It is two months ago that I have prepared dinner. In the evening, I am exhausted and I go to bed at eight”* (P1, female, aged 56)*.*

Many patients experienced a shift in their social relationships towards reduced outgoing activities and loss of contacts. Although many described their spouse or closest ones as very considerate, some still experienced social isolation; they did not feel understood, and they felt left alone.

#### The GP as a discussion partner

The GP was an important figure to all patients; both as a trustworthy authority and a partner for discussion that could help distinguish between symptoms and serious disease, and the GP also served as the gatekeeper to tests and investigations in the specialised healthcare system.

The patients contacted the GP when feeling sad or anxious, when needing reassurance due to new or intensified symptoms, or when doubting about the need for further investigations. More patients found it very helpful to talk to their GP and perceived their GP as accessible and attentive. A young woman described it this way:

*“I can go to my GP if I feel distressed. Then I just sit there and cry. I am really happy with her. She takes her time with me and yet makes sure that I get to see a specialist if she finds it relevant”* (P10, female, aged 20).

While the majority of patients voiced a satisfactory relationship with their GP and reported on continuously and supportive contacts, others described the relationship with their GP as an arena for struggle for acknowledgement and recognition of the need for further investigations.

### Domain 3: motivation

The motivation for self-management in patients with PPS was explored to identify factors of importance, including the use of an eHealth intervention.

#### Guidance from the GP

Guidance on how to manage the symptoms, especially guidance by the GP, was a key motivator for most of the patients. The patients found that being offered an eHealth programme by their GP would increase their motivation to use it. Nevertheless, although it was meant to be a self-help programme, some patients wished the GP to guide their activities in the programme and follow-up on progress. A young woman described it this way:

*“I think it would be nice if you could get a sort of homework in an online programme and then your GP would follow-up on whether you have done it or not”* (P10, female, aged 20).

The patients generally underlined the importance of the self-help programme being a part of their collaboration with their GP rather than an alternative to GP visits.

#### Need for hope

Some patients described a feeling of hopelessness concerning their situation. They worried about how their symptoms would affect their future, whether they would be able to continue work, take care of their family, and whether the symptoms would persist for their entire life. Some patients found that their GP and other healthcare professionals focussed overly much on acceptance and learning to live with symptoms instead of seeking the cause for the symptoms and a way to fix them.

Many patients expressed a need for seeing other patients with PPS to learn how they successfully managed their daily life. A young women described it this way:

*“It would be really helpful if you could get a feeling of not being alone by talking to or seeing others in the same situation and see how they manage their symptoms”* (P3, female, aged 23).

More patients suggested a forum where they could meet peers, or alternatively watch videos or case stories presenting other people living with PPS and introducing their management strategies. It was of utmost importance to the patients that the focus of an eHealth programme was on offering hope, positive experiences and effective strategies.

#### Tracking of symptoms

The patients found it very helpful to complete the BDS checklist prior to the interview. The symptom registration provided them with a visible and precise overview of their symptoms. They suggested the BDS checklist to be used for tracking and monitoring of symptoms. As described by a woman in her early thirties:

*“It is really difficult to tell how you are doing from day to day. Therefore, it is really helpful to have this checklist to monitor whether you are going in the right direction”* (P9, female, aged 32).

One patient suggested that the BDS checklist could be completed prior to seeing the GP. This would help the patient to better prepare for the consultation and to actively participate in the decision-making process. A male patient described how it had been helpful for him to present a symptom registration list to his GP. Then he did not have to constantly pay attention to and remember the symptoms to be able to account for every one of them when visiting the GP. He continued:

*“It is a relief to have presented all symptoms to my GP. Now, it is up to him to conclude on my condition. I have done my part; the rest is his responsibility”* (P5, male, aged 39).

Consequently, the BDS checklist could potentially serve as a ‘space to park symptoms’, a shared point of departure and a tool for monitoring. Yet, the list could also serve as a medium for transferring the responsibility from the patient to the GP.

#### Easy access to self-help strategies

In their everyday life, the participating patients applied a wide variety of more or less useful self-help strategies in search for symptom relief and improved quality of life. Hence, they seemed highly motivated for taking responsibility. Yet, they lacked guidance to navigate ‘the jungle of management strategies’. As PPS were rarely introduced as a reasonable explanation for their symptoms, guidance on this was rarely provided in the consultation with the GP.

Many of the patients expected that a self-help programme prescribed by their GP would be a safe and easy way to improve their self-management of PPS. They assigned a high degree of trust in their GP and thus in the content of such a programme. One patient put it this way:

*“If she [the GP] could tell me to visit this webpage to get information on my symptoms and their cause, then I wouldn’t have to use the internet so much; each time [you do an internet search for symptoms], you feel as if you are going to die. Instead, I would know that somebody has worked hard and carefully to create this programme, and that they know what they are talking about as opposed to an internet doctor”* (P10, female, aged 20).

### Themes identified in the GP interviews

The GP interviews provided information on PPS from the perspective of the healthcare professional. The three domains in the COM-B model were used to identify the GPs’ needs in terms of providing support for patients with PPS to self-manage their symptoms.

### Domain 1: capability

Capability was explored in order to establish whether the GPs’ felt physically and psychologically capable of supporting patients with PPS in the self-management of their symptoms.

### Physical capability

#### Missing biomedical reassurance

The six GPs participating in the study viewed their primary role as a GP to be to diagnose, treat and reassure their patients. This role was challenged by patient with PPS. Although the GPs were often fairly sure that no serious underlying disease caused the symptoms, the patients were often referred to further tests and investigations in the specialised healthcare system. Sometimes such referral was seen as a means to support the assessment made by the GP. At other times, it was to reassure the patient:

*“And I have to admit that sometimes you refer the patient to further tests at the hospital even though you have a clear expectation that everything is normal. But when blood tests and a scan confirm this, the patient is reassured to a higher extent”* (GP 2).

However, when symptoms persist, negative test results may prompt further healthcare seeking and initiate an Odyssey of referrals and investigations. Most of the GPs were aware of this dilemma and reflected on the challenges of the missing biomedical reassurance. While GPs with a special interest in PPS felt confident in their ability to identify PPS and provide an adequate symptom explanation to their patients, others expressed a need for more useful tools to support the patients’ understanding and acceptance of PPS.

### Psychological capability

#### Dealing with uncertainty

All GPs described diagnostic uncertainty as a condition tightly knotted to the frontline of healthcare, specifically to patients with PPS. A GP explained how she always conducts biomedical tests to be sure that symptoms are not explained by any well-defined physical disease prior to introducing PPS as a possible explanation:

*“I do not feel a huge degree of uncertainty when it comes to patients with PPS. However, we have to hedge our bets because we are so afraid of missing severe illness in the patients. Therefore, I always start with biomedical investigations”* (GP1).

The GPs had different opinions about expressing their uncertainty to the patients. A GP argued that she sometimes shared her thoughts with the patient when she made a referral to further physical investigations. She would then state that she did not expect any critical findings and that the investigations were done only to err on the side of caution. Another GP explained that he dealt with the diagnostic uncertainty by drawing on his many years of experience:

*“When you have been a GP for almost 20 years, you have a certain experience and then you are coping with diagnostic uncertainty, and you are also able to express your uncertainty to the patient. I often say to these patients that I am not sure what is causing your symptoms, but I am sure, based on the findings, that it is nothing severe”* (GP5).

The GPs used different strategies for handling the diagnostic uncertainty of PPS. While some of the GPs did not introduce PPS until every other potential relevant diagnosis was excluded, others introduced it as a potential explanation equal to physical and psychological explanations and prior to completed diagnostic work-up.

#### Timing in bringing PPS into play

Although the introduction of PPS was handled differently by the participating GPs, they all agreed on the importance of bringing PPS into play early in the symptom trajectory to avoid unnecessary tests and investigations.

Yet, some of the GPs expressed difficulties as to the early introduction of the concept of PPS. They described barriers related to worries of being mistaken but also to how the patient would react. A GP explained:

*“What you need is to acknowledge the patient and introduce it with respect. I don’t think that is very difficult […] But I think there are barriers to both the patient and to us; sometimes we are afraid of how the message will be received by the patient and whether we will get dismissed by him or her”* (GP 1)*.*

Thus, besides the fear of overlooking serious disease, reluctance to bring PPS into play was also caused by fear of harming the patient-physician relationship and the risk of being rejected.

### Domain 2: opportunity

The GPs’ physical opportunity was explored to understand how the environment and the resources in general practice affect the GPs’ support of patients with PPS in the self-management of symptoms. Social opportunity was explored to understand how the cultural norms and the social cues affect the way GPs think about and manage patients with PPS.

### Physical opportunity

#### Lack of resources

The GPs considered consultations with patients with PPS to be time-consuming in general. The patients often present with a variety of multiple, vague and diffuse symptoms that need further exploration and elaboration and that do not fit the time-restricted consultations. Hence, the GPs described a lack of time, and some requested more specific and easily accessible tools for managing patients with PPS. The perceived lack of options for treatment and management of PPS made some of the GPs adopt a cautious attitude towards these patients:

*“I think that I omit patients with PPS because we have only few options to help these patients. And because of that, I feel much more hesitant”* (GP6).

Frustration and feelings of inadequacy were often related to a primarily biomedical approach. A GP explained:

*“As a GP, you really want to diagnose and reassure the patient satisfactorily; they come into my practice with the expectation that I will be able to tell them what is the matter and offer them the needed treatment. However, when it comes to patients with PPS, I have neither the time, nor the tools to help them sufficiently”* (GP2).

GPs taking a special interest in PPS or psychotherapy mentioned time as a barrier to adequate management of patients with PPS. However, these GPs explicitly considered PPS in a broader perspective, including the patients’ circumstances of life and the balancing of physical and psychological factors as potential contributors to symptoms. Importantly, these GPs did not express the same level of need for new tools or treatment options. Instead, they provided a multi-factorial illness explanation and used the management tools at hand; two used activity and symptom registrations, and all of them used continuity and follow-up appointments as an active management strategy. A GP explained:

*“I think that one way to manage these patients is to offer them some follow-up appointments and just talk with them”* (GP6).

Thus, apart from lack of time, the GPs’ perceived lack of resources seemed to be greatly influenced by their interest in and knowledge of PPS.

### Social opportunity

#### Cultural norms in the healthcare system

Some GPs expressed powerlessness when it came to patients with PPS. These patients were found to be challenging due to the lack of a biomedical explanation for symptoms and the perceived lack of treatment options combined with a highly biomedical approach in the healthcare system. In general, the GPs requested closer and better collaboration with the specialised healthcare system. One of the GPs described patients with PPS as ‘no one’s responsibility’, making it the business of the GP. This made some GPs feel ‘home alone’ with the patient, which led to frustration and exhaustion.

Other GPs critically examined their own role as collaborators. They thought they should be better at preparing their colleagues in the specialised healthcare system, e.g. by an explicit statement in their referrals that no positive findings are expected, but the investigation is needed in order to be sufficiently sure. From their point of view, this approach might prompt their colleagues to touch upon the issue of PPS and not simply return the patient to their GP with recommendations on additional investigations. For this to happen, they deemed it necessary to share their thoughts with the patient prior to the referral. One of the GPs said:

*“I think it’s a huge problem that we keep examining the patients because none of us dare say that we don’t expect a physical explanation for their symptoms. Instead, the patients become more and more nervous as we all keep searching for an answer that isn’t there*” (GP4).

Thus, from the perspective of the GPs, the norm associated with a narrow biomedical focus leads to an inadequate collaboration and little clarity of roles. The patient will often be bounced back and forth between general practice and specialised healthcare. A GP concluded:

*“The more we* [*healthcare professionals*] *act in compliance with each other, the more reassured the patient gets, and the safer we feel as physicians”* (GP4).

### Domain 3: motivation

We explored factors important for GPs' motivation to support patients with PPS to self-manage their symptoms through an eHealth programme.

### Fewer and more beneficial consultations

Almost all of the GPs agreed that being supportive to their patients made their professional life meaningful and fulfilling, especially when contributing to clarification and improved function and quality of life. Thus, the GPs were highly motivated by the idea of providing patients with easy access to self-management tools for PPS. One GP said:

*“My challenge is that, as a physician, I cannot do much for these patients. Currently, I don’t have any proper service to offer them. Therefore, a self-help programme would be welcome”* (GP2).

Several GPs suggested that a self-help programme should include tailored information and tangible illness explanations, including support to the selection of management strategies that could be helpful for the individual patient. They saw a potential for a synergistic effect between the self-help programme and their encounters with the patient. A GP suggested:

*“Our collective agreement allows for a range of talk therapies. These could be used in connection with a self-help programme […] There are great perspectives in providing the patient with homework that afterwards can be shared with his or her GP”* (GP5).

Yet, not all GPs aligned with the idea of close collaboration around a self-help programme. A few GPs were primarily motivated by the notion that a self-help programme might relieve the pressure on the GP, e.g. by reducing patients’ need for consultations. Thereby, it was thought to benefit both the patient and the GP.

#### Better prepared consultations

Some of the GPs believed that an online self-help programme with an integrated symptom screening tool might help the patient to better prepare for consultations. Furthermore, they believed that such a programme could potentially be helpful to the GP, who could initiate a dialogue with the patient based on the data provided by the programme. A GP proposed:

*“I think that it would be a good idea to let the patient know that he or she is expected to interact with the programme as part of the treatment, and that a report will be sent to the GP every three, six or nine months […] Then I would know what has happened in the programme when the patient comes to see me”* (GP 3).

The GPs were generally worried about the increasing work load in general practice and saw a great potential in an online symptom screening tool to be filled in prior to the consultation. A GP explained:

*“I think that it seems a really good thing with this type of self-screening that focuses on how they are doing, and I don’t need to be involved in this part. But I can see that it works really well for many of my patients. This thing about how many steps do I take, how am I doing today, how is my pain and so on. Then they are able to monitor their own progress”* (GP 4).

It was hoped that such a tool would allow more time for dialogue in the consultation and that a self-help programme would have the potential to improve the quality of the patient-physician encounter and thereby lead to improved management of patients with PPS.

## Discussion

To the best of our knowledge, this is the first study to address the needs of patients and GPs prior to the development of an eHealth self-help programme for PPS in general practice. The findings highlight that certain aspects of capability, opportunity and motivation should be considered in the design and development of such a self-help programme. The main themes identified in the patient and GP interviews were comparable, and they focused on the timing of addressing the concept of PPS and on the subsequent understanding and explanation of symptoms, including diagnostic uncertainty. Furthermore, this study identified important motivational elements for an eHealth programme. These included making up for time restraints in the consultation, focusing on the patients’ need for hope, and making the GP available to guide the overall process.

The *capability* domain showed a gap between the patients’ and the GPs’ explanatory models in the consultation. Even though the patients clearly had concerns regarding severe disease, most of them were aware that other factors could explain their symptoms, which was documented in an earlier study [27]. However, they still initially presented physical symptoms in the consultation. The majority of GPs tended to use a biomedical approach to rule out serious disease and sometimes referred patients to physical investigations in secondary care (defensive biomedical approach). This dynamic seemed to downgrade psychological and social factors and delay the introduction of PPS in the initial consultations, even though patients and GPs agreed on the advantage of bringing these issues into play early on.

The main reason for not bringing PPS into play earlier in the consultation was uncertainty in both patients and GPs. *Dealing with uncertainty* and *timing in bringing PPS into play* were central themes in both patient and GP interviews. The patients were worried and wanted to be sure that their symptoms were not caused by severe disease. Likewise, the GPs feared overlooking severe disease and agreed that it was important to first conduct biomedical investigations before addressing differential diagnoses such as PPS. GPs have been found to face difficulties in recognising and labelling PPS; even when no somatic problem is indicated, many GPs are uncertain and afraid of overlooking serious disease [28]. In a systematic critical review, Alam et al. claimed that diagnostic uncertainty is a routine inevitability in general practice due to the undifferentiated symptoms presented to GPs [29].

Our findings on *capability* showed a need among both patients and GPs to acquire improved knowledge and skills with regard to their understanding of PPS, communicating about PPS and dealing with uncertainty. Some of the GPs in our study argued that they were reluctant to introduce PPS as they did not have any valid treatment to offer. Thus, the perception that the main task of the GP is to diagnose, treat and reassure the patient became a barrier to adequately managing PPS. Henningsen stated that good communication with the patients is essential at all stages of PPS and levels of care, including reassurance, anticipation of likely outcomes of diagnostic tests, positive explanations of the functional character of the disorder and motivation of the patient to actively engage in the coping with PPS [13]. However, our findings showed that PPS is often not communicated in the consultation, and management strategies are not properly addressed, leaving the patients to seek alternatives for symptom relief and management. Many patients felt lost in *the jungle of management strategies* and requested more specific guidance from their GP.

Studies have shown that patients’ need for support and compassion are not met by GPs’ provision of somatic screening and interventions [30, 31]. Salmon et al. stressed that patients with somatoform disorders feel satisfied and empowered by medical explanations that are tangible and involving. Empowering explanations may have the potential to improve patients’ wellbeing and to reduce high healthcare demands [32]. For the GPs, participation in the present study meant that they had to explain the term PPS to the invited patients. Therefore, the patient inclusion became a tool for the GP to initiate a conversation about PPS using a biopsychosocial frame, which several of the included patients found helpful. This highlights the potential of bringing PPS into play earlier in the consultation process in an easy and understandable manner.

Under the *opportunity* domain*, lack of resources* was an issue for the GPs, especially for patients with PPS as these consultations were found to be time-consuming. Furthermore, the GPs described *cultural norms in the healthcare system* to cause a general feeling of frustration towards patients with PPS. This causes patients with PPS to be referred continuously to examinations in search of the missing biomedical explanation, leaving the patients in No Man’s land. The patients described that they often used their *GP as a partner for discussion*. However, they were also aware of, and to some degree restrained by, the limited resources in general practice and requested more time to talk to the GP and discuss the symptoms. The limited time of practice consultations in Denmark are per se a barrier to patients with multiple and unspecific symptoms such as PPS. Likewise, Houwen et al. concluded that the communication in consultations on MUS could be improved if the GPs pay more attention to the patients’ agenda, and if the GPs prepare their consultations and focus on the issues that matters most to the patients. Furthermore, they suggest that GPs should be honest with patients when they do not understand the origin of symptoms [12]. However, our study pointed out that GPs are reluctant to share their diagnostic uncertainty with the patient.

In our exploration of *opportunity* issues with the patients, it became clear that the patients needed support on how to involve their social network in the management of PPS since they experienced a *shift in social roles due to PPS*. They missed seeing peers and other patients to get motivated on how to manage PPS in their daily life. The importance of a social network for maintaining good health is also described by Holt-Lunstad, Smith and Layton [33]. Likewise, the patients in our study expressed a great *need for hope.* By having the opportunity to follow other patients’ good examples, the patients believed they would gain more hope for the future and become more motivated to self-manage their persistent symptoms. These findings are in line with previous studies, which have found hope to be vital to recovery and to be cultivated by supportive interpersonal relationships, including thoughtful interaction and facilitative communication with healthcare professionals [34, 35].

Self-help interventions for PPS may be associated with reduced symptom severity and improved quality of life [19]. We discussed motivational factors relevant to an online self-help programme for persistent symptoms with both patients and GPs. We found *need for hope, guidance from the GP, tracking of symptoms* and *easy access to self-help strategies* to be important motivational factors. The patients were generally motivated to self-manage their symptoms, but the GP had an important role in guiding the patients in the right direction. Although face-to-face contact is essential to both patients and GPs, the GPs were motivated to use an eHealth programme if it could result in *fewer and beneficial consultations* and *better prepared consultations*. The GPs’ motivation for using a self-help intervention for patients with PPS was primarily to support the patients in their own environment and to avoid the patients’ dependence on continuous contact to the GP. This motivation was partly due to the well-known negative attitude of many GPs towards patients with PPS and GPs’ perceived lack of effective management strategies [14]. Both patients and GPs articulated that better preparation of the patients for the consultations could help create a better starting point for communication about PPS.

### Strengths and limitations

The use of qualitative interviews in this study allowed for in-depth understandings of both the patients’ and the GPs’ experiences with and opinions on dealing with PPS to understand their needs.

The use of the COM-B model as a theoretical framework for both the interview guides and the final mapping of subthemes facilitated the identification of themes to be addressed in future eHealth interventions for PPS. Furthermore, this use highlighted areas that need to be taken into consideration when eHealth programmes are implemented in general practice. However, the majority of interventions guided by the COM-B model have aimed to change lifestyle behaviours, including diet and physical exercise, and only few studies have used the COM-B model within the bio-psycho-social area [36]. Nevertheless, we found the use of the COM-B model helpful to guide the research process and to ensure that themes relevant to behaviour change were explored.

The study was conducted by a cross-disciplinary research group. The different academic backgrounds and the continuous discussions throughout the research process, from the development of the interview guide to the data analysis and the preparation of the paper, reduced the risk of preconceptions that could otherwise have comprised the results.

Despite these strengths, the study has some potential limitations that need to be considered. The transferability of the study results may have been hampered. The included GPs were recruited by purposeful sampling based on one of the author’s personal knowledge of the GPs. Although this author did not perform the interviews, this relationship might have caused the participating GPs to refrain from less socially acceptable statements. Furthermore, we included a rather small number of GPs. Yet, we believe that we succeeded in reaching data saturation; no new subthemes were revealed at the end of the analysis process, and we obtained rich and varying descriptions of GP attitudes towards and experiences with patients with PPS, including self-help interventions for this group of patients.

Another limitation was that many of the included patients had suffered from PPS for a long period of time. Thus, their attitudes and experiences may differ from those of patients with more recent symptom onset. Ideally, we should have included more patients with recent symptom onset to inform the development of the self-help programme as part of early treatment of PPS. However, as no firm diagnostic criteria for PPS exist, we depended on the GPs’ ability to identify the relevant patients. Thus, the included sample may reflect the GPs’ difficulties in recognising and identifying patients with PPS. This stresses the need for continuing education on PPS in general practice [37, 38].

The study contributed with new perspectives that should be considered in the design of eHealth programmes for PPS. Future exploration of patients’ and GPs’ experiences of feasibility, acceptability and usability of such eHealth programmes for PPS is needed to evaluate whether the findings from the current study can be successfully integrated into care.

## Conclusions

In conclusion, several unmet needs emerged from the in-depth interviews with patients with PPS and GPs. First, we identified a need to bring PPS into play early on in the process to take a more bio-psycho-social approach to the management of PPS. Second, both patients and GPs request better skills to communicate about PPS and to manage uncertainty. Third, the patients need hope when their symptoms persist. Finally, the patients want the GP to engage in their illness trajectory and need guidance from their GP on how to self-manage their PPS. These aspects should be given priority in future self-help programmes integrated into healthcare for patients with PPS in general practice.

## Supplementary Information

**Additional file 1.**

## Data Availability

The datasets generated during and/or analysed during the current study are not publicly available due to the highly sensitive nature of the topic under investigation and the transcripts in Danish, but the data are available from the corresponding author on reasonable request.

## Abbreviations

BDS: Bodily distress syndrome
COM-B: Capability, opportunity, motivation – behaviour
eHealth: Electronic health
GP: General practitioner
MUS: Medically unexplained symptoms
PPS: Persistent physical symptoms
