# Supplementary material for: Development of an eHealth programme for self-management of persistent physical symptoms: a qualitative study on user needs in general practice
Source: BMC Fam Pract. 2021 Feb 7;22:33. doi: 10.1186/s12875-021-01380-5 (PMC7869449; doi:10.1186/s12875-021-01380-5)
Supplement: Supplementary file 1 — Additional file 1. [file 12875_2021_1380_MOESM1_ESM.docx]

# Supplementary file 1

###### Interview guide - patients

| **Domains** | **Interview questions** |
| --- | --- |
| **Background information** | Would you start by telling a little about yourself (age, marital status, family, education, occupation, general health condition)?  Which considerations did you make when the GP asked you to participate in this project?  I would like to hear about your course: Which symptoms do you experience (BDS questionnaire)? When and how often? When did you first contact your GP about your symptoms, and what made you do it? How did your GP react? What happened next? What do you think about still experiencing symptoms (accept of condition)? How has it affected your life and your everyday activities?  Is there something in your course that you think could have been different? |
| **Capability**  Physical capability  Psychological capability | Would you tell a little about how you handle your symptoms in everyday life (exercise, rest, pain relief, stress relief, etc.)?  Try to describe your day as it was yesterday from when getting out of bed until getting back in bed in terms of your symptoms.  What makes it harder vs. easier for you to handle your symptoms? What works the best?  Do you feel that you have the resources to both physically and mentally handle your symptoms? What do you miss being able to do?  What influences your resources? Is there something that you need to be able to handle the symptoms (knowledge, energy, strength, perseverance, capabilities/skills)?  How do you communicate with others about your condition/situation? |
| **Opportunity**  Physical opportunity  Social opportunity | How do you articulate to others how you feel? How do you experience that your surroundings react? (colleagues, relatives, friends) (social support)? How have you handled the situation in terms of your work place/education? How did your work place/education handle it? Have you been in contact with the municipality because of your symptoms? How did you experience it? Is something missing in this connection?  Where do you seek help and support for handling your symptoms? Have you used groups on Facebook, the internet, networks or other resources to get help? |
| **Motivation**  Automatic motivation (habits)  Reflective motivation | Could you tell about some good or bad habits that you have had for handling your symptoms? How did they arise? Are there any habits that you would like to change? Which? What would motivate you to change them? Is there something that you miss in this connection?  Have your symptoms made you change anything in your life? What is important to you? What do you think about the future?  What do you think could be the cause of your symptoms?  (Do you think that your symptoms are there for a reason, e.g. that your body is trying to tell you something?) |
| **Technology** | Which types of technology do you use in your everyday life? For what and when?  What do you think about offering an internet-based programme for patients such as you? Pros and cons?  Based on our conversation, what do you find important to include in an internet-based self-help programme? Do you have any ideas of what such programme should include for you to see it as helpful in making you get better?  Which role should your own GP have in such internet-based programme? Should your GP, for example, be able to follow how much you are using it? Should you be able to communicate with your GP through the programme?  How do you think the programme is best introduced to patients like you?  What would seem motivating for using such programme? |

###### Interview guide - GPs

| **Domains** | **Interview questions** |
| --- | --- |
| **Background information** | Would you start by telling a little about yourself as a general practitioner (age, number of years as a practicing GP, special functions as a doctor, special interests)? |
| **Capability**  Physical capability  Psychological capability | How often do you see patients with persistent symptoms in your practice?  How do you experience them?  How do you handle them in the consultation? How and when do you articulate to them that their symptoms do not have a medical explanation? Which challenges do you experience?  Some doctors experience diagnostic uncertainty and ”bet on two horses”. Is this something that you recognise for patients with persistent symptoms? How do you handle it?  Is there something that could make it easier for you to handle such situation/uncertainty?  What do you recommend your patients to do to handle their persistent and unexplained symptoms? Are there any capabilities that you lack in this connection? What could help you? |
| **Opportunity**  Physical opportunity  Social opportunity | How do you experience the general approach in the healthcare system to this group of patients? How do you perceive the collaboration with the specialised system on this group of patients (primary sector, municipalities, work places, specialised healthcare system)?  How do you experience that your colleagues look at this group of patients?  Which needs do you see in the patients with persistent symptoms? Which opportunities do you have for helping them? Is there something you need in this regard? |
| **Motivation**  Automatic motivation (habits)  Reflective motivation | What motivates you in your daily life as a GP? What motivates you specifically to help patients with persistent symptoms?  What do you expect of patients with persistent symptoms? How do you communicate your expectations to them? |
| **Technology** | What is your attitude in general to referring your patients to a technological solution/a self-help programme? Pros and cons for you as a GP?  Based on our conversation, what do you find important to include in an internet-based self-help programme for patients with persistent symptoms to ensure that it makes sense to you as a GP? Do you have any ideas as to what such programme should include?  Which role should you have as a GP in the programme? Are there any skills that you lack in this connection?  How and when in the course do you think that the programme should be introduced to the patients? What could help you with this? Which physical barriers do you see in your practice for using an internet-based self-help programme for patients with persistent symptoms? (Should it be integrated into existing systems?)  Which organisational framework do you see as hampering and facilitating in general practice in connection with using internet-based self-help programmes for patients with persistent symptoms?  What would motivate you as a GP to use such programme in your practice? |
